# Susceptibility-guided vs. empirical 10-day quadruple treatment for *Helicobacter pylori*-infected patients: A prospective clinical trial of first-line therapy

**DOI:** 10.3389/fmicb.2022.973975

**Published:** 2022-09-07

**Authors:** Peiwei Li, Jing Jin, Yan Chen, Jianjuan Ma, Qin Du, Yuehua Han

**Affiliations:** Department of Gastroenterology, Second Affiliated Hospital, Zhejiang University School of Medicine, Hangzhou, China

**Keywords:** *Helicobacter pylori*, eradication, susceptibility-guided therapy, empirical therapy, randomized controlled trial

## Abstract

**Background:**

The increasing antimicrobial resistance of *Helicobacter pylori (H. pylori*) has resulted in a fall in cure rates. We aimed to assess the effectiveness of first-line susceptibility-guided therapy and furazolidone-based quadruple therapy for *H. pylori*-infected patients.

**Methods:**

Subjects with *H. pylori*-infection were randomly assigned to either 10-day susceptibility-guided treatment or empiric treatment in a 2:1 ratio. Susceptibility-guided therapy was based on susceptibility to clarithromycin, and patients with susceptible strains received clarithromycin 500 mg twice daily and otherwise minocycline 100 mg twice a day was administered. Patients in the empiric therapy group was treated with furazolidone 100 mg twice a day. During treatment, all patients were given esomeprazole 20 mg twice daily, colloidal bismuth pectin 200 mg twice daily, and amoxicillin 1 g twice daily.

**Results:**

A total of 248 patients were screened and 201 were finally included. Empiric and susceptibility-guided regimens were both successful with per-protocol eradication rates of 90.5% (57/63) vs. 88.5% (108/122) (*p* = 0.685) and intent-to-treat eradication rates of 85.1% (57/67) vs. 80.6% (108/134) (*p* = 0.435). No significant difference in eradication rates were observed among the furazolidone group, clarithromycin group and minocycline group.

**Conclusion:**

Both susceptibility-guided therapy and quadruple therapy containing furazolidone can achieve good eradication rates. For population with a high rate of resistance, quadruple therapy containing furazolidone and bismuth may be a more practical choice for first-line treatment.

## Introduction

Globally, *Helicobacter pylori (H. pylori*) infects approximately 4.4 billion people, making it one of the most prevalent pathogens in humans (Hooi et al., 2017). *H. pylori* is a leading cause of chronic gastritis, peptic ulcer, gastric mucosa-associated lymphoid tissue lymphoma and gastric cancer, and plenty of researchers have emphasized the eradication of *H. pylori* infection to reduce these diseases (Lee et al., 2013; Graham et al., 2017). Moreover, *H. pylori* has also been suggested to be a risk factor for many extra-gastrointestinal diseases, such as cardiovascular diseases (de Korwin et al., 2017; Wang et al., 2017). Thus, *H. pylori* eradication is important for public health. However, the widespread use of antibiotics has led to a rise in antimicrobial resistance, which has decreased the cure rate for *H. pylori* infections (Megraud et al., 2021).

Traditional therapy for infectious disease depends on local, regional, or patient-specific antimicrobial susceptibilities (Graham and Dore, 2016), and susceptibility-guided treatment should be the best strategy if available. The currently recommended first-line *H. pylori* therapy in China is bismuth quadruple therapy (Liu et al., 2018). One study reported that rates of *H. pylori* resistance in China for clarithromycin, metronidazole, and levofloxacin were 35.1, 82.7, and 46.9%, respectively (Chen et al., 2019). Due to China’s high antibiotic resistance rates, treatment regimens have become increasingly complex. Personalized treatments based on antibiotic susceptibility represent a novel therapeutic option. However, susceptibility testing of *H. pylori* is difficult to perform and not practical in many clinical setting. As the resistant rate of amoxicillin and furazolidone remains low, the bismuth quadruple therapy containing these two medicines has been proved to be effective in China (Qiao et al., 2021). Besides, randomized controlled trials have shown that a bismuth quadruple therapy containing tetracycline remained highly effective (Chen et al., 2016), while tetracycline is difficult to obtain in many areas including China. Fortunately, several studies have proved the efficacy of minocycline in *H. pylori* eradication, which is a tetracycline derivative (Song et al., 2016).

Here, we conducted a study to assess the resistance of drugs used in *H. pylori* eradication, and to compare the efficacy of susceptibility-guided therapy (containing clarithromycin or minocycline according to susceptibility testing) with empiric therapy containing furazolidone.

## Materials and methods

### Study design

This study was a prospective, interventional, open-label, single-center trial performed between 2019 and 2020 at the Second Affiliated Hospital of Zhejiang University, School of Medicine. Informed consent was obtained from all subjects and the trial was approved by the hospital’s Ethics Committee. It was registered in ResMan, a web-based medical research public management platform, and the registration number was ChiCTR2000038308. The study was also conducted in accordance with the Declaration of Helsinki, and the recommendations of the CONSORT statement for reporting randomized controlled trials. *H. pylori* infection was determined by urea breath test (^13^C-UBT or ^14^C-UBT) or histology. Subjects would be excluded if they were younger than 18 years of age, previously treated for *H. pylori*, pregnancy or lactation, previous gastric surgery, presence of significant clinical diseases or malignancy, use of antisecretory drugs, antibiotics or bismuth within the past 4 weeks, or allergy to any of the research drugs.

### *Helicobacter pylori* isolation and antimicrobial susceptibility testing

We collected two biopsy specimens (one from gastric antrum, and one from gastric corpus) during gastroscopy (260/290 series, Olympus, Tokyo, Japan) for *H. pylori* isolation. Under microaerophilic conditions (85% N_2_, 10% CO_2_, and 5% O_2_), the specimens were cultured and maintained on brain heart infusion agar medium (Oxoid, Basingstoke, United Kingdom) containing 5% defibrinated sheep blood at 37°C. *H. pylori* isolates were identified by colony morphology, microscopic image of Gram-negative helix-shaped bacterial morphology, and positive for urease, oxidase, and catalase.

The E-test method (AB Biodisk, Solna, Sweden) was applied to determine the minimum inhibitory concentrations (MICs). MIC values were determined after 72 h of incubation and we used *H. pylori* ATCC 43526 for quality control. Resistance to antibiotics was defined as follows: amoxicillin, MIC ≥0.5 μg/ml; clarithromycin, MIC >1.0 μg/ml; metronidazole, MIC >8 μg/ml; tetracycline, MIC >4 μg/ml; and levofloxacin, MIC >1 μg/ml.

## Intervention

Patients with *H. pylori* infection were randomly assigned to either 10-day susceptibility-guided treatment or empiric treatment in a 2:1 ratio. Technicians performing culture, antimicrobial susceptibility testing or urea breath test were blinded to treatment allocation. Patients in the empiric therapy was treated with furazolidone 100 mg twice daily for 10 days. Susceptibility-guided therapy was according to susceptibility to clarithromycin, and patients with susceptible strains received clarithromycin 500 mg twice daily and otherwise minocycline 100 mg twice a day was administered. During treatment, all patients were given esomeprazole 20 mg twice daily, colloidal bismuth pectin 200 mg twice daily, and amoxicillin 1 g twice daily.

At least 4 weeks after therapy completion, ^13^C- or ^14^C-urea breathe test was performed to assess *H. pylori* eradication, and negative urea breath test result was defined as eradication.

### Statistical analysis

We used intention-to treat (ITT) and per-protocol (PP) analysis to assess eradication rates. For the ITT analysis, all subjects were included, while only subjects who followed the protocol were included in the PP analysis. Patients without follow-up UBT were defined as treatment failures in the ITT analysis. Characteristics of the population and distribution of antibiotic resistance was performed using descriptive statistics. Student’s *t*-test was used for continuous data comparation, and chi-square test was applied for categorical data. All *P*-values were two−sided, and *P* < 0.05 was defined as statistically significance. All analyses were conducted using SPSS v.21 Statistics program.

## Results

**FIGURE 1:**
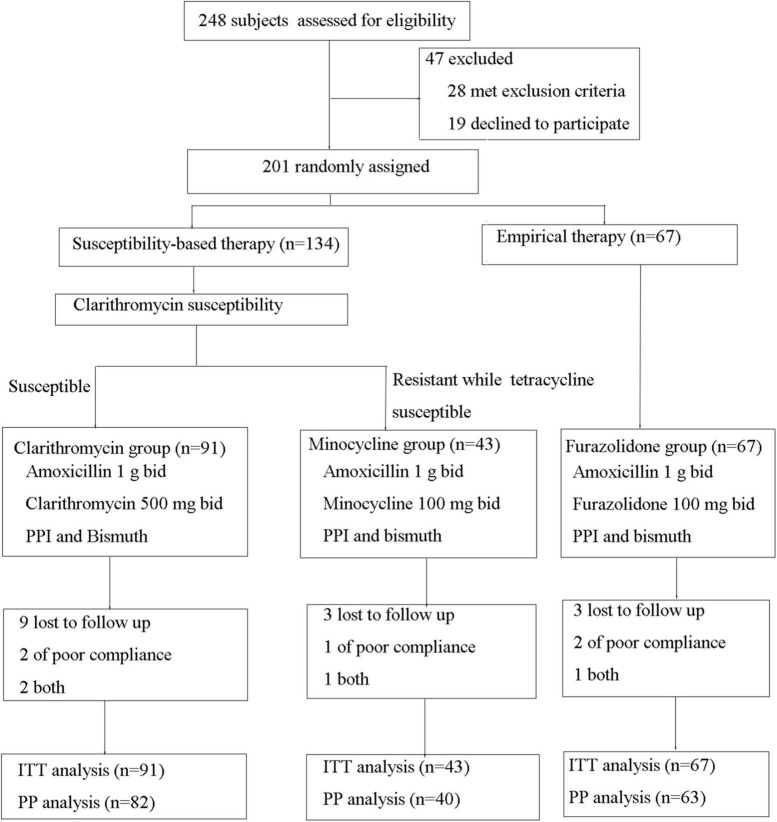
Flow diagram of the study.

A total of 248 *H. pylori*-infected patients were evaluated for eligibility, and 47 met exclusion criteria or declined to participate and were excluded. Finally, 201 patients were enrolled and divided into furazolidone group (*n* = 67) and susceptibility-guided therapy group (*n* = 134) (as shown in Figure 1). The mean age was 42.5 for furazolidone group and 45.0 for susceptibility-guided therapy group (*p* = 0.180) (Table 1). There were 28 males in furazolidone group (41.8%) and 59 males in susceptibility-guided therapy group (44.0%) (*p* = 0.760) (Table 1). Overall, 15 patients were lost to follow-up UBT and five patient had poor compliance (four both had poor compliance and lost to follow-up), which were defined as treatment failure in the ITT analysis and were not included in the PP analysis. A total of 185 patients were finally included in the PP analyses (Figure 1).

**TABLE 1:** Baseline characteristics of the included patients.

	Susceptibility-guided therapy group (*n* = 134)	Empiric therapy group (*n* = 67)	*P*-value
Age (mean)	45.0	42.5	0.180
Gender (M/F)	59/75	28/39	0.760
**Antibiotic resistance *n* (%)**			
Clarithromycin	44 (32.8%)	14 (29.2%)	0.640
Metronidazole	101 (75.4%)	27 (56.3%)	0.013
Levofloxacin	44 (32.8%)	10 (20.8%)	0.118
Tetracycline	3 (2.2%)	0 (0%)	0.296
Furazolidone	4 (3.0%)	0 (0%)	0.226
Amoxicillin	0 (0%)	0 (0%)	–

### Results for antimicrobial susceptibility testing

Among the 201 included subjects, culture was not performed for nine subjects and were failed for another nine patients (in the furazolidone group), and thus 183 subjects were included in the antimicrobial susceptibility test. The rate of metronidazole resistance was highest (70.0%, 128/183), followed by clarithromycin (31.7%, 58/183) and levofloxacin (29.5%, 54/183), furazolidone (2.2%, 4/183) and tetracycline (1.6%, 3/183). There were no cases of amoxicillin resistance. Metronidazole resistance rate was higher in the susceptibility-guided therapy group (75.4%) compared with furazolidone group (56.3%) (*p* = 0.013), while no significant difference in resistance rate of other antibiotics was observed (Table 1).

In the susceptibility-guided therapy group, 43 were resistant to clarithromycin and were included in the minocycline group and the remaining 91 were included in the clarithromycin group.

### *Helicobacter pylori* eradication rates and safety

As shown in Table 2, PP analysis indicated that the eradication rates were 90.5% (57/63) in the furazolidone group and 88.5% (108/122) in the susceptibility-guided therapy group. No significant difference between the two groups was found (*p* = 0.685). Moreover, there was no significant difference between furazolidone group (90.5%), clarithromycin group (89.0%, 73/82), and the minocycline group (87.5%, 35/40) (*p* = 0.892).

**TABLE 2:** Eradication rate of each group in intention-to treat (ITT) and per-protocol (PP) analysis.

	Empiric therapy group	Susceptibility-guided therapy group	Clarithromycin group	Minocycline group
ITT analysis	85.1% (57/67)	80.6% (108/134)	80.2% (73/91)	81.4% (35/43)
PP analysis	90.5% (57/63)	88.5% (108/122)	89.0% (73/82)	87.5% (35/40)

All *P* > 0.05.

Intention-to treat analysis also suggested no significant difference in eradication rates between furazolidone group (85.1%, 57/67) and the susceptibility-guided therapy group (80.6%, 108/134) (*p* = 0.435). There was no significant difference between furazolidone group (85.1%), clarithromycin group (80.2%, 73/91), and the minocycline group (81.4%, 35/43) (*p* = 0.727) (Table 2). Adverse effects were similar with furazolidone group (7.5%, 5/67) and the susceptibility-guided therapy group (6.7%, 9/134) (Table 3), and no severe adverse effects were observed.

**TABLE 3:** Adverse effects of the included patients.

	Susceptibility-guided therapy group	Empiric therapy group
Adverse effects	9 (6.7%)	5 (7.5%)
Taste alteration	2 (1.5%)	1 (1.5%)
Skin rash	1 (0.7%)	2 (3.0%)
Abdominal pain	3 (2.2%)	0 (0%)
Fever	0 (0%)	1 (1.5%)
Nausea and vomiting	2 (1.5%)	1 (1.5%)
Fatigue	2 (1.5%)	1 (1.5%)

All *P* > 0.05.

## Discussion

In the current study including 201 patients, the resistant rate of metronidazole was high (70.0%), followed by clarithromycin (31.7%) and levofloxacin (29.5%), while the resistant rate was low for furazolidone (2.2%), tetracycline (1.6%), and amoxicillin (0%). The resistant rate of metronidazole and clarithromycin appeared to be higher than a previous study conducted in Korea, which suggested that resistant rate of metronidazole and clarithromycin was 29.5 and 17.8%, respectively (Lee et al., 2019). However, the resistant rate was similar with another study performed in China, of which the resistance rates of *H. pylori* for clarithromycin, levofloxacin, metronidazole, amoxicillin, and furazolidone were 26.12, 28.69, 96.79, 0, and 0%, respectively (Pan et al., 2020). The high rates of resistance to antibiotics have significantly reduced eradication rate of *H. pylori* (Sugano et al., 2015; Liu et al., 2018). As reported, traditional therapy eradication rate for *H. pylori* is below 80% in many cities, especially in high-risk areas for antibiotic resistance (Graham et al., 2014; Sebghatollahi et al., 2018).

Theoretically, therapy basing on the results of susceptibility testing for infectious diseases should be recommended, which is associated with higher efficacy, fewer side effects and unnecessary antibiotic use. According to the Maastricht V/Florence Consensus Report, susceptibility-guided therapy has been recommended after the second-line treatment fails (Malfertheiner et al., 2017). In a previous meta-analysis, tailored therapy was found to be more effective than empirically chosen treatment for eradicating *H. pylori* (Gingold-Belfer et al., 2021). The meta-analysis included both first-line and rescue treatments, and the role of susceptibility-guided therapy in first-line *H. pylori* remains unclear. Several studies suggested higher efficacy of susceptibility-guided therapy compared with empirically chosen treatment, while other studies found inconsistent results. Besides, the availability, accuracy, and cost-effectiveness of susceptibility-guided therapy should be considered in the clinical practice of *H. pylori* eradication (Matsumoto et al., 2019).

As a nitrofuran antibiotic, furazolidone damages bacterial DNA and interferes with normal bacterial metabolism. The primary and secondary resistance to furazolidone is low for *H. pylori*, and quadruple therapy containing furazolidone has been widely used in China (Xie et al., 2018). According to guidelines, furazolidone is recommended for eradication of *H. pylori* due to the low resistance (Malfertheiner et al., 2017; Liu et al., 2018). A number of studies have explored the efficacy and safety for rescue therapy and for naïve *H. pylori*-infected patients (Fakheri et al., 2001; Liang et al., 2013; Qiao et al., 2021). As reported by Liang et al. (2013) treatment regimens containing furazolidone were significantly more effective than treatments without furazolidone in rescue therapy of *H. pylori*. Another study compared clarithromycin with furazolidone for naïve *H. pylori*-infected patients, and recommended furazolidone-based quadruple therapy because of the high eradication rate, excellent cost-effectiveness and acceptable safety (de Korwin et al., 2017). In China, bismuth potassium citrate, colloidal bismuth pectin, and colloidal bismuth subcitrate are widely available. Currently, bismuth-containing quadruple therapy is the first-line treatment for *H. pylori* infection because it is effective against both susceptible and resistant strains (Malfertheiner et al., 2017; Liu et al., 2018).

In the current study, we compared the efficacy of susceptibility-guided therapy with empirical quadruple therapy containing furazolidone and bismuth, and both PP and ITT analyses suggested that these two therapies were comparable (90.5% in the furazolidone group and 88.5% in the susceptibility-guided therapy group for PP analysis; 85.1% in the furazolidone group and 80.6% in the susceptibility-guided therapy group for ITT analysis). Moreover, in the susceptibility-guided therapy group, no significant difference was found between clarithromycin group and the minocycline group. We observed a low rate of adverse effects for both furazolidone group (7.5%) and the susceptibility-guided therapy group (6.7%), and no severe adverse effects were found. The results further supported the use of furazolidone and minocycline in *H. pylori* eradication, and supported quadruple therapy containing furazolidone and bismuth when susceptibility test was unavailable.

There were several limitations in the current study. First, this was a single-center randomized controlled trial, which may limit generalizing the results. Second, obtaining tetracycline and minocycline remains difficult in many areas of China. Third, routinely performing *H. pylori* antimicrobial susceptibility test is difficult in most areas, which limits the use of susceptibility-guided therapy.

In conclusion, both susceptibility-guided therapy and empirical quadruple therapy containing furazolidone can achieve good eradication rates. For population with a high rate of resistance, empirical quadruple therapy containing furazolidone and bismuth may be a more practical choice for first-line treatment.

## Data availability statement

The raw data supporting the conclusions of this article will be made available by the authors, without undue reservation.

## Ethics statement

The studies involving human participants were reviewed and approved by the Ethics Committee of the Second Affiliated Hospital of Zhejiang University, School of Medicine. The patients/participants provided their written informed consent to participate in this study.

## Author contributions

YH and PL designed and conceived this study, and prepared for the manuscript. JJ, YC, and JM collected clinical samples and performed the experiments. QD, YH, and PL analyzed the data. All authors contributed to the article and approved the submitted version.
